# Lower prevalence of *Blastocystis* sp. infections in HIV positive compared to HIV negative adults in Ghana

**DOI:** 10.1371/journal.pone.0221968

**Published:** 2019-09-03

**Authors:** Veronica Di Cristanziano, Rossella D´Alfonso, Federica Berrilli, Fred Stephen Sarfo, Maristella Santoro, Lavinia Fabeni, Elena Knops, Eva Heger, Rolf Kaiser, Albert Dompreh, Richard Odame Phillips, Betty Norman, Torsten Feldt, Kirsten Alexandra Eberhardt

**Affiliations:** 1 Institute of Virology, University of Cologne, Faculty of Medicine and University Hospital of Cologne, Cologne, Germany; 2 Department of Systems Medicine, University of Rome Tor Vergata, Rome, Italy; 3 Department of Clinical Sciences and Translational Medicine, University of Rome Tor Vergata, Rome, Italy; 4 Kwame Nkrumah University of Science and Technology, Kumasi, Ghana; 5 Komfo Anokye Teaching Hospital, Kumasi, Ghana; 6 National Institute for Infectious Diseases L. Spallanzani—IRCCS, Rome, Italy; 7 Kumasi Center for Collaborative Research in Tropical Medicine, Kumasi, Ghana; 8 Clinic of Gastroenterology, Hepatology and Infectious Diseases, University Hospital Düsseldorf, Düsseldorf, Germany; 9 Department of Tropical Medicine, Bernhard Nocht Institute for Tropical Medicine and I. Department of Medicine, University Medical Center Hamburg-Eppendorf, Hamburg, Germany; Central University of Tamil Nadu, INDIA

## Abstract

**Background:**

Sub-Saharan Africa is endemic for intestinal parasites and distinguished for the largest burden of HIV cases. *Blastocystis* sp. is one of the most common protists infecting humans but its role in human disease is still controversial. Aim of this study was to investigate the prevalence of *Blastocystis* sp. in HIV positive and negative adults in Ghana and its association with immune status and other risk factors.

**Methods:**

122 HIV positive outpatients and 70 HIV negative blood donors from the Komfo Anokye Teaching Hospital in Kumasi, Ghana, were included in the present study. Demographic, clinical and laboratory data were collected and HIV positive patients distinguished for CD4+ T cell count <200 cells/μl (n = 54) and >200 cells/μl (n = 68). A *Blastocystis*’s phylogenetic analysis was performed to determine sample subtype (ST).

**Results:**

The prevalence of *Blastocystis* sp. in adult HIV positive individuals was lower than in HIV negative persons (6.6% vs. 20.0%, p = 0.008) and *Blastocystis* sp. ST1 was the most prevalent strain. Within HIV positive participants, the prevalence of *Blastocystis* sp. was lower in those individuals with CD4+ T cell count <200 cells/μl than in patients with higher CD4+ T cell count (1.9% vs. 10.3%, p = 0.076). Multiple regression analysis revealed that *Blastocystis* sp. was inversely associated with an obese Body Mass Index (BMI) in HIV negative persons (p = 0.040). Presence of *Blastocystis* sp. was correlated with higher CD4+ T cell count in HIV positive participants (p = 0.049).

**Conclusion:**

It is largely reported that people living with HIV (PLHIV) in Africa are affected from parasite infections and that co-infections may adversely impact on their immune status, accelerating progress to AIDS and worsening gastrointestinal manifestations. Differently, in this study *Blastocystis* sp. was associated with a better immune status jointly with a healthy body weight while it seems to be reduced with the progression of HIV infection. This data agree with recent suggestions that *Blastocystis* sp. can represent a component of the healthy gut microbiota.

## Introduction

*Blastocystis* sp. represents worldwide one of the most common human intestinal protozoan parasites, showing prevalence higher than 5% in developed countries and much higher in developing countries [1]. However, the morphological diversity exhibited by this parasite and the lack of standardization in diagnostic techniques have led to confusion about its prevalence and role as human pathogen [2]. Originally, *Blastocystis* sp. was considered a commensal microorganism of the gastrointestinal tract. However, the increasing detection rate, largely attributable to the utilization of improved molecular methods, has raised the question as to whether *Blastocystis* sp., or different *Blastocystis* sp. subtypes, could be implicated in gastrointestinal disorders and therefore redefined as a potentially pathogenic symbiont (i.e. pathobiont).

Hence, the current knowledge on *Blastocystis* sp. is characterized by disagreement about its clinical relevance, pathogenic potential, and need of treatment, particularly in immunocompromised patients, such as subjects affected by HIV/AIDS [3–5]. Beside asymptomatic infections, the spectrum of symptoms associated with *Blastocystis* sp. includes nausea, abdominal cramps, flatulence and acute or chronic diarrhea [6, 7]. The manifestations in *Blastocystis* sp. carriers could be related to genetic differences on the subtype (ST) level [8, 9]. Indeed, the *Blastocystis* sp. sequences exhibit remarkable genetic diversity and at least 17 subtypes, among which the first nine found in humans with and without intestinal disorders, have been described based on the gene coding for the small-subunit ribosomal RNA (ssu rRNA) [10]. *Blastocystis* sp. ST1-ST4 account for 90% of all human carriage, with ST3 and ST1 appearing to be the most common subtypes [11, 12]. ST3 is reported to be the most frequent subtype detected in symptomatic patients, followed by ST1 and ST2 [8]. In Sub-Saharan Africa provision of safe water supplies, sanitation and hygiene are often inadequate and constitute risk factors that facilitate infections with feco-oral transmissible agents [13]. Furthermore, Sub-Saharan Africa counts for the majority of HIV and AIDS cases reported worldwide [14, 15].

As known, diarrhea is a major cause of morbidity in HIV-infected patients, occurring in 30–60% of AIDS patients in developed countries and in up to 90% in developing countries [14, 16, 17]. In Ghana, 300,000 people are estimated to be living with HIV/AIDS and only 70,000 of them are reported to be having access to antiretroviral therapy (ART) [18]. Recent studies have shown that the presence of parasitic infections could disturb the balance of anti-HIV immune responses and contribute to HIV replication which could accelerate progression to AIDS [19].

The aim of the present study was to deepen our understanding of the implications of *Blastocystis* sp. detection in persons with and without HIV-infection living in Ghana. For this purpose, we studied the differences in genotype of *Blastocystis* sp. and the association with the immune status, clinical symptoms, therapeutic treatments, and intestinal co-infections.

## Material and methods

### Study design and study population

Between November 2011 and November 2012, consecutive adult HIV positive patients presenting to the HIV outpatient Department of the Komfo Anokye Teaching Hospital in Kumasi, Ghana, and HIV negative blood donors from the same hospital were recruited for a prospective observational cohort study on clinical and sociodemographic determinants of *H*. *pylori* co-infection among HIV-infected and non-infected individuals [20, 21]. To analyse the prevalence, risk factors and clinical implications of *Blastocystis* sp. co-infection, participants were randomly selected from HIV positive patients with CD4+ T cell counts below or more than 200 cells/μl, and from HIV negative individuals from the above mentioned observational cohort. One stool sample from each participant was tested for *Blastocystis* sp. and other common enteric pathogens.

This study was carried out in accordance with the World Medical Association’s Declaration of Helsinki. The protocol was approved by the Committee on Human Research of the Kwame Nkrumah University of Science and Technology in Kumasi, Ghana: CHRPE/AP/12/11, and the ethics committee of the Medical Council in Hamburg, Germany: PV3771. Written informed consent was obtained from all participants before enrolment.

### Data collection and laboratory methods

Demographic and clinical data were collected by trained study personnel using a standardized questionnaire. Blood samples were collected and the analysis of CD4+ T cell count was performed in Ghana using a FACSCalibur flow cytometer (Becton Dickinson, Mountain View, California). Aliquots of native stool samples were freshly frozen and stored at -80°C before being transported to Germany on dry ice.

700 μl of a 10% suspension in Phosphate-Buffered-Saline (PBS) of each stool sample was prepared and nucleic acids were extracted from this suspension by using the automated platform VERSANT kPCR Molecular System and the VERSANT Sample Preparation 1.0 Reagents Kit (Siemens Healthcare Diagnostics), according to the manufacturer’s instructions.

### *Blastocystis* sp. detection and subtyping

In order to detect the presence of *Blastocystis* sp., the small subunit rRNA (SSU-rDNA) 600 bp fragment amplification was carried out by using primers RD5—BhRDr while a second amplification was performed using the primers Blasto 2F and Blasto 2R according to the protocol as previously described [22, 23]. The PCR amplicons, after visualization by gel electrophoresis, were purified and directly sequenced on both strands by the Bio-Fab Research (Rome, Italy).

A phylogenetic analysis on *Blastocystis* sp. Sanger sequences was performed to determine subtype and genetic variability between patients and subtypes. Briefly, the sequences were aligned with reference sequences of the nine *Blastocystis* sp. subtypes found to date in human (ST1-ST9). The alignment was edited using the BioEdit program version 7.0.5.3. Tree was generated using the Generalised Time Reversible (GTR) substitution model and 1,000 bootstrap replicates with maximum-likelihood (ML) method using RAxML program (https://github.com/stamatak/standard-RAxML) [24]. Pairwise genetic distances between referenced *Blastocystis* sp. subtypes and patient subtypes were generated using the Tajima-Nei model [25]. All positions with less than 95% site coverage were eliminated. Therefore, fewer than 5% alignment gaps, missing data, and ambiguous bases were allowed at any position. The variation rate among sites was modelled with a gamma distribution (shape parameter = 0.5). Evolutionary analyses were conducted using MEGA6.

### Co-infections with other enteric pathogens

In order to exclude intestinal disorders caused by other enteric pathogens, all *Blastocystis* sp. positive samples were screened for the presence of common pathogens. The total nucleic acids were extracted as previously described [26]. For each specimen, the molecular diagnostic xTAG GPP Luminex assay (Luminex Molecular Diagnostics, Toronto, Canada), including adenovirus types 40/41, norovirus genogroup I and II (GI/GII), group A rotavirus, *Campylobacter spp*., *Clostridium difficile* toxin A/B, *Escherichia coli* O157, enterotoxigenic *Escherichia coli* (ETEC) LT/ST, *Salmonella spp*., Shiga-like toxin producing *E*. *coli* (STEC) stx1/stx2, *Shigella spp*., *Vibrio cholerae*, *Yersinia enterocolitica*, *Cryptosporidium hominis* and *C*. *parvum*, *Entamoeba histolytica*, *Giardia duodenalis*, and the multiplex FTD Viral gastroenteritis (Fast-Track Diagnostics, Luxembourg), including noro-, adeno-, rota-, astro-, and sapovirus, were utilized.

### Statistical analysis

Continuous variables were expressed as mean ± standard deviation (SD) or median (interquartile range, IQR) and compared using the unpaired Student’s t-test or the Wilcoxon rank sum test. Proportions were compared using either the χ^2^ test or the Fisher exact test, as appropriate. Multiple logistic regression models were used for assessing independent associations between demographic, medical and immunological parameters and *Blastocystis* sp. status. Two-sided p-values were presented and statistical significance was determined at α = 5%. Statistical analyses were conducted using R version 3.5.1 (R Foundation for Statistical Computing, Vienna, Austria).

## Results

### *Blastocystis* sp. prevalence

**Fig 1:**
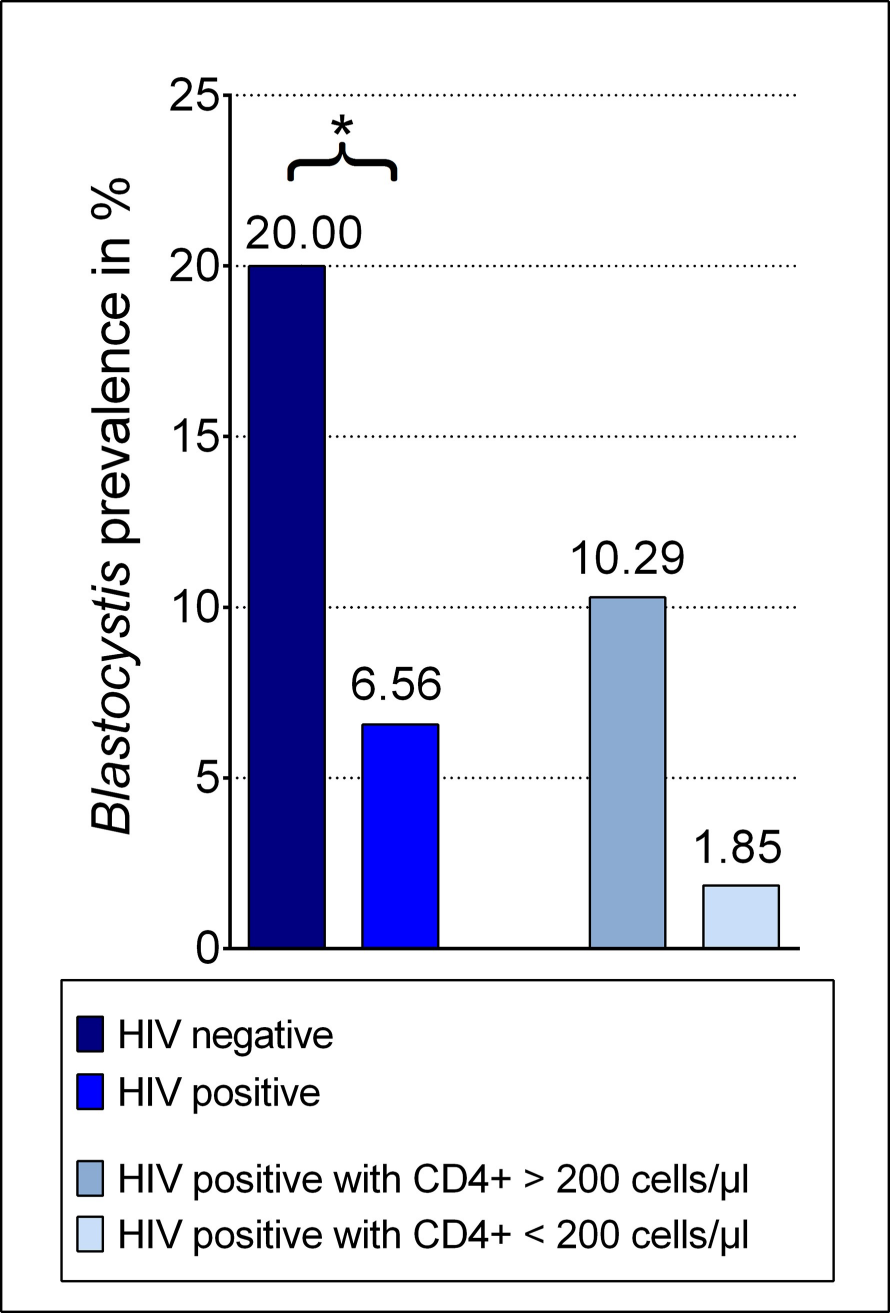
Prevalence of *Blastocystis* sp. Prevalence of *Blastocystis* sp. (%) in HIV negative compared to HIV positive persons, and HIV positive persons with CD4+ T cell count higher than 200 cells/μl, compared to HIV positive persons with CD4+ T cell count less than 200 cells/μl.

122 participants with HIV infection (n = 54 with CD4+ counts < 200 and n = 68 with CD4+ counts > 200 cells/μl) and 70 HIV negative individuals were enrolled from the Komfo Anokye Teaching Hospital in Kumasi. The prevalence of *Blastocystis* sp. in non-HIV infected adult participants seen was 20.0% (n = 14/70), while the rate of co-infections with *Blastocystis* sp. in HIV positive persons was significantly lower (6.6%, n = 8/122, p = 0.008, Fig 1). A tendency, although not statistically significant, mark for a lower prevalence of *Blastocystis* sp. in HIV positive subjects with CD4+ T cell count of less than 200 cells/μl than in patients with higher CD4+ T cell count (1.9% vs. 10.3%, p = 0.076).

### Phylogenetic analysis

**Fig 2:**
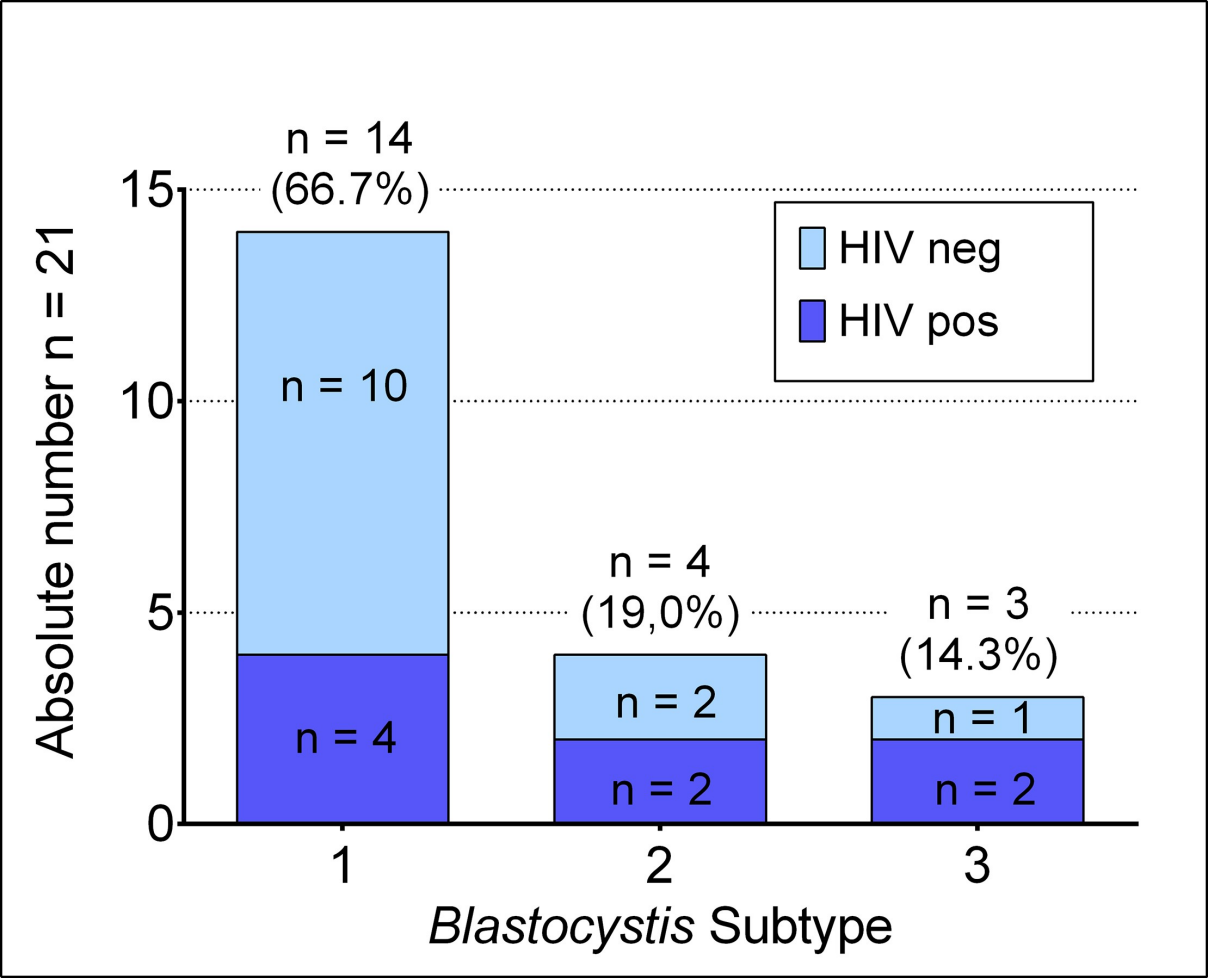
*Blastocystis* sp. subtype distribution.

Out of the 22 *Blastocystis* sp. positive patients, 21 corresponding sequences were successfully obtained. For one HIV negative patient, no valid sequence was obtained. Phylogenetic analysis revealed that ST1 was the most prevalent strain (Fig 2). In particular, 14 patients were infected with ST1 strain (66.7%), 4 patients with ST2 (19.0%), and 3 infected with ST3 (14.3%).

### Characteristics of *Blastocystis* sp. positive and negative individuals

No differences related to sociodemographic factors were observed between *Blastocystis* sp. positive and negative participants within the two subgroups of HIV positive and negative persons (Table 1). However, *Blastocystis* sp. positive persons without HIV infection showed lower CD4+ T cell counts [869 (IQR 783–984) vs 1051 (IQR 915–1360), p = 0.025] and more frequently a normal body weight as compared to *Blastocystis* sp. negative persons (Body Mass Index (BMI) between 18.5 and 25 kg/m^2^, p = 0.016). Moreover, HIV positive patients infected with *Blastocystis* sp. had higher median CD4+ T cell counts than those without *Blastocystis* sp. co-infection [527 (IQR 367–651) vs 264 (IQR 110–489), p = 0.035].

**Table 1:** Comparison of sociodemographic and medical parameters of *Blastocystis* sp. positive and negative participants within subgroups of HIV positive and HIV negative participants.

Parameters	HIV positive subjects			HIV negative subjects		
	Total	*Blastocystis* sp. positive	*Blastocystis* sp. negative	Total	*Blastocystis* sp. positive	*Blastocystis* sp. negative
**n (%)**	122	8 (6.6)	114 (93.4)	70	14 (20.0) ^**§**^	56 (80.0)
**Age in years, mean ± SD**	40.3 ± 8.8	37.9 ± 12.9	40.5 ± 8.5	34.2 ± 13.1 ^**§**^	36.6 ± 18.3	33.6 ± 11.5
**Female gender, n (%)**	89 (73.0)	7 (87.5)	82 (71.9)	43 (63.8)	9 (64.3)	34 (63.0)
**CD4+ T cell count/μl, median (IQR)**	289 (113–496)	527 (367–651)	264 (110–489) *	1007 (859–1261) ^**§**^	869 (783–984)	1051 (915–1360) *
**Gastrointestinal symptoms, n (%)**	14 (11.5)	1 (12.5)	13 (11.4)	19 (29.2)	3 (21.4)	16 (31.4)
**BMI (kg/m**^**2**^**), n (%)**				^**§**^		*
Low (≤18.5)	17 (14.2)	0 (0)	17 (15.18)	1 (1.7)	1 (8.33)	0
Normal (>18.5-≤25)	76 (63.3)	5 (62.5)	71 (63.39)	30 (52.6)	9 (75,00)	21 (46.67)
High (>25-≤30)	27 (22.5)	3 (37.5)	24 (21.43)	26 (45.6)	2 (16.67)	24 (53.33)
**Occupation, n (%)**				^**§**^		
Housewife	1 (0.82)	0 (0)	1 (0.88)	3 (4.69)	0 (0)	3 (6.00)
Farmer	10 (8.20)	1 (12.50)	9 (7.90)	2 (3.13)	1 (7.14)	1 (2.00)
Trader	70 (57.38)	4 (50.00)	66 (57.90)	25 (39.06)	5 (35.71)	20 (40.00)
Salary worker	8 (6.56)	0 (0)	8 (7.02)	18 (28.13)	3 (21.43)	15 (30.00)
Unemployed	13 (10.66)	3 (37.50)	10 (8.77)	2 (3.13)	0 (0)	2 (4.00)
Other	20 (16.39)	0 (0)	20 (17.54)	14 (21.88)	5 (35.71)	9 (18.00)
**Education, n (%)**				^**§**^		
Primary school	24 (19.67)	2 (25.00)	22 (19.30)	8 (12.50)	0 (0)	8 (16.00)
Junior high school	56 (45.90)	6 (75.00)	50 (43.86)	6 (9.38)	1 (7.14)	5 (10.00)
Senior high school	14 (11.48)	0 (0)	14 (12.28)	35 (54.69)	9 (64.29)	26 (52.00)
Tertiary Institution	3 (2.46)	0 (0)	3 (2.63)	9 (14.06)	3 (21.43)	6 (12.00)
No formal education	25 (20.49)	0 (0)	25 (21.93)	6 (9.38)	1 (7.14)	5 (10.00)
**Access to tap water, n (%)**	64 (52.5)	4 (50.0)	60 (52.6)	40 (60.6)	9 (64.3)	31 (59.6)
**Electricity in the household, n (%)**	109 (89.34)	7 (87.50)	102 (89.47)	62 (93.94)	14 (100.00)	48 (92.31)
**Fridge/Freezer in household, n (%)**	84 (68.9)	5 (62.5)	79 (69.3)	50 (75.8)	12 (85.7)	38 (73.0)

*p<0.05 for within group comparisons (*Blastocystis* sp. positive/negative)

^**§**^p<0.05 for between group comparisons (total HIV positive/ total HIV negative)

BMI = Body Mass Index.

### Co-infections

13 (59%) of all 22 *Blastocystis* sp. positive participants were co-infected by other gastrointestinal pathogens. Gastrointestinal symptoms were only reported by four participants co-infected with other pathogens (Table 2) and of these, only one was HIV positive. Almost all of *Blastocystis* sp. positive individuals with other gastrointestinal co-infections (n = 11, 91.67%) were infected by *Blastocystis* sp. ST1. In contrast, only one third of *Blastocystis* sp. positive participants without other pathogens were carrying ST1 (33.33%, p = 0.009). Detected co-infections were by adenovirus, ETEC, STEC, *Shigella* spp., *Salmonella* spp., and norovirus GII (single infection n = 12, multiple infections n = 1).

**Table 2:** Comparison of medical parameters, symptoms and subtypes of *Blastocystis* sp. infected participants with and without other gastrointestinal co-infections detected.

Parameters of *Blastocystis* sp. positive subjects	Intestinal co-infections present, n = 13	Intestinal co-infections absent, n = 9
**Age in years, mean ± SD**	36.2 ± 18.5	38.2 ± 13.3
**HIV positive, n (%)**	3 (23.08)	5 (55.56)
**CD4+ T cell count/μl in HIV positive, median (IQR)**	385 (260–495)	583 (470–791)
**CD4+ T cell count/μl in HIV negative, median (IQR)**	869 (746–958)	901 (810–1008)
**BMI (kg/m**^**2**^**) in HIV positive**	22.3 (21.9–22.6)	25.1 (23.9–28.7)
**BMI (kg/m**^**2**^**) in HIV negative**	21.7 (20.9–22.5)	23.2 (21.9–28.0)
**ART intake in HIV positive, n (%)**	1 (33.33)	2 (40.00)
**Co-trimoxazole intake in HIV positive, n (%)**	2 (15.39)	2 (22.22)
**Gastrointestinal symptoms, n (%)**	4 (30.77)	0 (0)
**Feverish, n (%)**	3 (30.00)	1 (16.67)
**Cough, n (%)**	4 (30.77)	1 (11.11)
**Other gastrointestinal pathogens detected, n (%)**		NA
Adenovirus	6 (46.15)	NA
ETEC	5 (38.46)	NA
STEC	1 (7.69)	NA
Shigella	2 (15.38)	NA
Norovirus GII	1 (7.69)	NA
Salmonella	1 (7.69)	NA
***Blastocystis* sp. subtypes (ST), n (%)**		*
ST1	11 (91.67)	3 (33.33)
ST2	1 (8.33)	3 (33.33)
ST3	0 (0)	3 (33.33)

*p<0.05

ART = Antiretroviral Therapy; BMI = Body Mass Index; NA = Not available

In our previous work, we found the prevalence of *H*. *pylori* infection to be inversely correlated with the degree of immunosuppression, a relation that was similarly seen for *Blastocystis* sp. infections in the present study. However, the prevalence of *H*. *pylori* was not different in *Blastocystis* sp. positive and negative participants within the HIV positive and negative subgroups (62.5% vs 54.9%, p = 0.731 in HIV-positives, and 92.9% vs. 88.2%, p = 0.621 in HIV-negatives) and comparable to our previous findings in the full cohort (51.5 and 88.0%) [20].

### HIV-related characteristics of *Blastocystis* sp. positive and negative individuals

Although CD4+ T cell counts differed significantly between HIV positive persons with and without *Blastocystis* sp. co-infection, no statistically significant differences in time since diagnosis of HIV-infection, intake of antiretroviral treatment, time since initiation of antiretroviral therapy, co-trimoxazole prophylaxis or rifampicin intake during the last 6 months were detected between them (Table 3). All but one patients on ART were receiving first-line therapy, either Zidovudine or Tenofovir with Lamivudine and either Efavirenz or Nevirapine and there was no correlation between drug combinations and *Blastocystis* sp. status observed in our data.

**Table 3:** Comparison of parameters related to HIV-infection between *Blastocystis* sp. positive and negative HIV positive participants.

	HIV positive (n = 122)	
Parameters	*Blastocystis* sp. positive (n = 8)	*Blastocystis* sp. negative (n = 114)
Time since diagnosis of HIV infection in months, mean ±SD	36.5 ± 39.77	24.26 ± 31.41
ART intake, n (%)	3 (37.50)	51 (44.74)
Time since initiation of ART in months, mean ±SD	63.67 ± 28.22	40.11 ± 26.47
Co-trimoxazole intake, n (%)	4 (50.00%)	35 (30.70)
Rifampicin intake, n (%)	2 (28.57)	10 (9.90)
Intake of other antibiotics, n (%)	0 (0)	1 (0.88)

ART = Antiretroviral Therapy

### Factors associated with *Blastocystis* sp. infection

In the group of HIV positive *Blastocystis* sp. positive participants the CD4+ T cell count was the only risk factor positively associated with *Blastocystis* sp. co-infection in the simple logistic regression model (Table 4). Also when adjusting (aOR) for other potential risk factors, such as gender, age, BMI, and current treatment in our multiple logistic regression model, *Blastocystis* sp. co-infection remained significantly associated with higher CD4+ T cell counts (aOR 1.22, 95% CI 1.00–1.50, p = 0.049).

**Table 4:** Factors associated with the presence of *Blastocystis* sp. in HIV positive and negative persons.

	HIV positive, n = 122		HIV negative, n = 70	
	Simple regression model	Multiple regression model	Simple regression model	Multiple regression model
Variable	OR (96% CI)	aOR (96% CI)	OR (96% CI)	aOR (96% CI)
**Age in years/10**	0.69 (0.27–1.60)	0.87 (0.36–1.94)	1.18 (0.75–1.80)	1.24 (0.70–2.30)
**Gender**				
Female	1	1	1	1
Male	0.37 (0.02–2.17)	0.53 (0.02–4.30)	0.94 (0.26–3.14)	0.68 (0.14–2.99)
**BMI (kg/m**^**2**^**)**				
< 25	1	1	1	1
>25	2.20 (0.43–9.63)	1.36 (0.20–7.66)	0.18 (0.02–0.76) *	0.17 (0.02–0.78) *
**CD4+ T cell count in count/μl /100**	1.20 (1.00–1.43) *	1.22 (1.00–1.50) *	0.82 (0.65–0.99)	0.86 (0.65–1.09)
**Receiving ART**			NA	NA
No	1	1	NA	NA
Yes		0.56 (0.09–3.19)	NA	NA
**Intake of Rifampicin**			NA	NA
No	1	1	NA	NA
Yes	2.57 (0.13–18.29)	2.95 (0.13–30.24)	NA	NA
**Intake of Co-trimoxazole**			NA	NA
No	1	1	NA	NA
Yes	2.26 (0.51–10.05)	2.22 (0.43–11.60)	NA	NA

*p<0.05

BMI = Body Mass Index; ART = Antiretroviral Therapy; NA = Not available

The simple logistic regression model revealed that a BMI >25 was inversely associated with *Blastocystis* sp. infection in the HIV negative group. Also after adjusting for gender, age and CD4+ T cell count in a multiple logistic regression model, higher BMI values were inversely correlated with *Blastocystis* sp. harboring (aOR 0.17, 95% CI 0.02–0.78, p = 0.040).

## Discussion

The prevalence of intestinal parasite infections reaches up to 95% in HIV positive persons in developing countries. These infections are caused both by protozoa and helminths and their main clinical manifestation is diarrhoea [14]. *Blastocystis* sp. is one of the most common intestinal protozoa infecting humans [3]. Data on the prevalence of *Blastocystis* sp. in Africa are scarcely documented and influenced by the method of detection. Our recent survey evidenced an overall prevalence of 58% in people living in a southern department of Côte d´Ivoire [23]. Similar high prevalence based on molecular analysis was reported from Liberia (70%) and from Senegal (100%) [10, 27].

The lack of available data on the potential association of the high circulation of *Blastocystis* sp. and the high prevalence of HIV infections in these geographical areas spurred us to consider whether *Blastocystis* sp. could have an impact on HIV positive patients that are more vulnerable to parasitic infections.

If on the one hand, several investigations have been performed to define the impact of parasitic enteropathogens on HIV patients in endemic areas already, on the other hand most of them focused on the main “known” pathogenic enteric parasites, while the role of *Blastocystis* sp. was marginally considered [28–32]. Although these studies give varying and, sometimes contradictory, results in respect to *Blastocystis* sp. infections in HIV/AIDS patients concerning its prevalence and related occurrence of clinical manifestations, this parasite is still considered an opportunistic cause of diarrhoea also in these individuals [3, 4].

In the present study, an unexpected result was the overall prevalence of *Blastocystis* sp., which was lower as compared to our previous data in a different country in West Africa and by similar molecular approach [23]. The more contained presence of *Blastocystis* sp. in the present cohort could be explained by the fact that more than half of the study participants were having access to tap water, 90% were having electricity in their household and around 70% stated owning a fridge [20, 21]. Furthermore, patients enrolled in this study, as HIV+ or blood donors were normally stimulated in the outpatient clinic for safeguard good health by good hygienic practices and were regularly controlled for health status. The age of subjects could also contribute to explain this result, given that our present cohort did not include children, who are more exposed to risk of infections in areas with poor hygiene conditions [33, 34].

Considering that adenovirus was the most frequent co-pathogen detected in ST1 positive patients, that adenovirus infections are mostly been thought to be species-specific, and that ST1 is also found frequently in humans, it might be hypothesized that inter-human transmissions play a major role in our cohort. The simultaneous use of two different commercial multiplex molecular assays allowed optimizing pathogen testing. The 6 adenovirus positive samples were detected by FTD assay only. Indeed, FTD Viral Gastroenteritis enables the detection of all 57 accepted human adenovirus types, whereas Luminex assay target adenovirus 40 and 41 only, which are the most frequent types associated with acute gastroenteritis. The discordant result about the norovirus GII positive specimen seem to confirm the lower reliability of Luminex assay for norovirus detection compared to other platforms, which could depend on higher complexity of xTAG GPP [35]. On the other hand, Luminex assay can detect a broad spectrum of clinically relevant enteric pathogens, including viruses, bacteria, and parasites.

Remarkably, HIV negative patients showed a rate of *Blastocystis* sp. infection significantly higher than HIV positive patients (20.0% vs 6.6%, p = 0.008) in agreement with what was found in China [36]. Similar results were observed in a study by Assefa et al. for protozoa other than *Blastocystis* sp. in HIV positive and negative subjects from Ethiopia [30].

Even more interesting, within HIV positive subjects, the prevalence of *Blastocystis* sp. was higher in those individuals with CD4+ T cell counts of more than 200 cells/μl than in patients with lower CD4+ T cell counts (10.3% vs. 1.9%, p = 0.076). Intake of medication, time since diagnosis of HIV infection or time since initiation of ART did not differ between *Blastocystis* sp. positive and negative persons co-infected by HIV. This data can be considered in agreement with results obtained in a study from Cameroon by Nsagha and colleagues [37].

Additionally, *Blastocystis* sp. was inversely associated with overweight in HIV negative persons, also after adjusting for confounders (p = 0.016 and p = 0.040). The only *Blastocystis* sp. positive patient with lean weight in the HIV negative subgroup was co-infected with *Salmonella* spp., which likely caused the reported gastrointestinal complaints of this individual. Also in a recent study on 316 individuals from Denmark and Spain, a tendency of *Blastocystis* sp. to be more common in lean individuals was observed [38]. Similarly, a higher *Blastocystis* sp. prevalence in normal weight individuals compared with overweight and obese ones was evident in the recent review by Beghini et al. [39]. However, it is well recognized that ART represents a strong risk factor for obese body weight in PLHIV and thus, it might be an explanation for not observing an association between a healthy body weight and *Blastocystis* sp. carriage in our HIV positive subgroup, but instead a clear correlation between BMI and CD4+ T cell count (p = 0.005) [40]. On the other hand, we found a significant association between a healthier immune status and *Blastocystis* sp. in these patients. The linkage between ART, obesity, CD4+ T cells and elevated levels of inflammatory markers which are also adversely correlated with progression of HIV disease is known [41]. Taking these findings together is reasonable to hypothesize that *Blastocystis* sp. could be related to a good health status without causing clinically harmful effects, as evidenced by the observed association with healthy body weight in HIV negative participants and a good immune status in HIV positive patients.

However, the observed association of *Blastocystis* sp. with lower CD4+ T cell counts and BMI values in HIV negative adults may implicate an immunomodulatory and potential adverse effect of this protist [42]. Thus, the increase of CD4+ T cells in *Blastocystis* sp. positive persons with HIV infection could result from a cell-mediated immune response towards the parasitic infection.

From the identification of the ST, our results appear in agreement with all those was already found in the African continent characterized by a higher prevalence of ST1-ST3 and an uncommon presence of ST4, the latter not detected in our cohort.

The analysis of clinical manifestations revealed some interesting aspects on the pathogenicity and clinical significance of this intestinal protist. A low number of *Blastocystis* sp. positive individuals, including only one HIV positive person, were symptomatic but all were detected co-infected by other pathogens. Recently, Beghini et al. suggested that *Blastocystis* sp. is a component of the healthy gut microbiome [39]. A similar association between *Blastocystis* sp. and eubiosis derived from a previous investigation which highlighted a different combination of microorganisms in gut microbiota of subjects infected by different intestinal protozoa [43]. Andersen et al. proposed a similar hypothesis using a metagenomics approach [44] and Audebert et al. found a higher bacterial alpha diversity in the faecal microbiota with the presence of *Blastocystis* sp. [45].

The present study provides some first data on the prevalence of *Blastocystis* sp. in Ghana and contributes to the debate on the impact of this parasite in human disease. An association of the presence of *Blastocystis* sp. with a better immune status jointly with a healthier body weight as shown in our study highlights the potential role of *Blastocystis* sp. as component of the healthy gut microbiota. This study has some limitations. Although the study design enables to detect potential associations of *Blastocystis* sp. with the immune status, clinical symptoms, therapeutic treatments, and intestinal co-infections, we cannot conclude on causalities. Longitudinal studies would be required to observe alterations of immune parameters according to *Blastocystis* sp. status, particularly after new infection with this protist. Moreover, the more intense application of advanced molecular approaches, including multiplexed assays and next generation sequencing, especially to stool samples coming from endemic areas, will provide further explanation regarding the clinical significance of *Blastocystis* sp. in the future.

## Supporting information

S1 File Prevalence of Blastocystis infections in Ghana dataset: Data_Di_Cristanziano_et_al. (XLSX)
