# Correction: Lower prevalence of *Blastocystis* sp. infections in HIV positive compared to HIV negative adults in Ghana

**DOI:** 10.1371/journal.pone.0224000

**Published:** 2019-10-11

**Authors:** Veronica Di Cristanziano, Rossella D´Alfonso, Federica Berrilli, Fred Stephen Sarfo, Maristella Santoro, Lavinia Fabeni, Elena Knops, Eva Heger, Rolf Kaiser, Albert Dompreh, Richard Odame Phillips, Betty Norman, Torsten Feldt, Kirsten Alexandra Eberhardt

There is an error in affiliation 6 for the author Lavinia Fabeni. The correct affiliation 6 is: Department of Experimental Medicine, University of Rome "Tor Vergata", Rome, 00133, Italy.
